# Traditional Chinese medicine Zhusha Anshen Wan: protective effects on liver, kidney, and intestine of the individual drugs using ^1^H NMR metabolomics

**DOI:** 10.3389/fphar.2024.1353325

**Published:** 2024-01-29

**Authors:** Dalu Wang, Chong Yu, Beixing Liu, Haifeng Wang

**Affiliations:** ^1^ Department of General Surgery, Shengjing Hospital of China Medical University, Shenyang, Liaoning, China; ^2^ Department of Traditional Chinese Materia Medica, Shenyang Pharmaceutical University, Shenyang, China; ^3^ Department of Pathogenic Biology, School of Basic Medical Science, China Medical University, Shenyang, China

**Keywords:** metabonomics, ^1^H NMR spectroscopy, detoxification, cinnabar, intestinal flora

## Abstract

**Introduction:** Zhusha Anshen Wan (ZSASW) is a traditional Chinese medicine compound mainly composed of mineral drugs. In clinical practice, ZSASW did not show the toxicity of administering equal doses of cinnabar alone, suggesting that the four combination herbs in ZSASW can alleviate the damage of cinnabar. The effect of each herb on reducing the toxicity of cinnabar has not been fully explained.

**Methods:** In our study, we utilized a metabonomics approach based on high-resolution 1H nuclear magnetic resonance spectroscopy to investigate the reduction of toxicity by each herb in ZSASW. Liver, kidney and intestinal histopathology examinations and biochemical analysis of the serum were also performed.

**Results:** Partial least squares-discriminant analysis (PLS-DA) was conducted to distinct different metabolic profiles in the urine and serum from the rats. Liver and kidney histopathology examinations, as well as analysis of serum clinical chemistry analysis, were also carried out. The metabolic profiles of the urine and serum of the rats in the CGU (treated with cinnabar and *Glycyrrhiza uralensis* Fisch) and CCC (treated with cinnabar and *Coptis chinensis* French) groups were remarkably similar to those of the control group, while those of the CRG (treated with cinnabar and *Rehmannia glutinosa* Libosch) and CAS (treated with cinnabar and *Angelica sinensis*) groups were close to those of the cinnabar group. The metabolic profiles of the urine and serum of the rats in the CGU and CCC groups were remarkably similar to those of the control group, while those of the CRG and CAS groups were close to those of the cinnabar group. Changes in endogenous metabolites associated with toxicity were identified. *Rehmannia glutinosa*, *Rhizoma Coptidis* and *Glycyrrhiza uralensis* Fisch could maintain the dynamic balance of the intestinal flora. These results were also verified by liver, kidney and intestinal histopathology examinations and biochemical analysis of the serum. The results suggested that

**Discussion:** The metabolic mechanism of single drug detoxification in compound prescriptions has been elucidated. *Coptis chinensis* and *Glycyrrhiza uralensis* serve as the primary detoxification agents within ZSASW for mitigating liver, kidney, and intestinal damage caused by cinnabar. Detoxification can be observed through changes in the levels of various endogenous metabolites and related metabolic pathways.

## 1 Introduction

Mercurials such as cinnabar (96% as HgS) have been used in traditional Chinese medicines (TCMs) as tranquilizers (Wang et al., 2007; Liu et al., 2008a). Additionally, its toxicological effects have been repeatedly reported (Wei et al., 2008). Mercury (Hg) is well known for its toxicity; thus, the presence of Hg in traditional medicines is great important (Liu et al., 2008b). Cinnabar, also called as red mercury sulfide or mercurous sulfide is a naturally occurring mineral that has been used in traditional medicine for centuries. It is available in various forms, such as powders, tablets, and capsules, and can be purchased online or at health food stores. Cinnabar is also used in some cosmetics and skin care products, although its use in these products is not always regulated by the FDA (Wang et al., 2013). Despite its long history of use in traditional medicine, cinnabar’s toxicity was not fully recognized until recently. The primary cause of cinnabar toxicity is the consumption of large amounts of cinnabar. This can occur through the ingestion of contaminated food or water or by inadvertently consuming an excessive amount of a product containing cinnabar. In addition, cinnabars can be absorbed through the skin when applied topically (Saper et al., 2008). The Chinese Pharmacopoeia Committee has reduced the allowable Hg or As content in traditional medicine by as much as 65% (Pharmacopoeia of China, 2010) (Zhou et al., 2009), but the mercury content in these cinnabar-containing prescriptions is still thousands of-fold greater than the allowable limits in Western countries. However, many reports have indicated that these prescriptions do not have toxic effects similar to those of minerals.

Zhusha Anshen Wan (ZSASW) is composed of cinnabar (*cinnabaris*) and *Coptis chinensis* French. (CC), *Angelica sinensis* (oliv.) Diels (AS), uncooked *Rehmannia glutinosa* Libosch. (RG), honey fried *Glycyrrhiza uralensis* Fisch. Zhusha Anshen Wan is a traditional Chinese medicine formula that has been used for centuries to address a range of conditions, such as insomnia, anxiety, and depression. The formula contains several ingredients, including Zhusha (cinnabar), Anshen (Ginkgo biloba), and other herbs (Zhou et al., 2010). It has been shown to have beneficial effects on regulating sleep patterns in rats with insomnia (Li, 2008) and to enhance the calming effects of pentobarbital (Gao et al., 2008). Cinnabar is the primary ingredient in Zhusha Anshen Wan and is thought to have sedative and calming effects on the mind and body. It is also thought to improve blood circulation and decrease inflammation (Wang et al., 2013), ZSASW was significantly less toxic than cinnabar, indicating that the four combined herbs in ZSASW could alleviate the injuries induced by cinnabar. However, the effects of each herb on reducing the toxicity of cinnabar have not been fully explained.

Omic field techniques have been important methods in the field of systems biology. Metabonomics is a branch of systems biology that involves the quantitative measurement of the dynamic multiparametric metabolic responses of living systems to pathophysiological stimuli or genetic modifications (Robertson, 2005). Currently, metabonomics has been utilized in therapy monitoring, pharmaceutical discovery, and the assessment of drug efficacy and toxicity (Keun, 2006). This strategy aligns well with the integrative and systemic features of TCM and has been selected by several researchers to study Chinese medicine syndrome patterns (Buriani et al., 2012). ^1^H NMR metabonomics is a technique used to investigate the metabolic changes that occur in cells and tissues following exposure to toxic substances. The analysis of the metabolites present in a sample involves nuclear magnetic resonance (NMR) spectroscopy. The main advantage of ^1^H NMR metabonomics over other methods is its noninvasive nature, which enables the analysis of biological samples without causing any damage or alteration to the tissue. Additionally, metabolomics can be used to identify various types of metabolites, such as amino acids, sugars, lipids, and organic acids, which can offer valuable insights into the toxicity of a substance. Indeed, a metabonomic approach has been applied to studies of toxicity related to the use of natural medicines such as Hei-Shun-Pian and its toxic component aconitum alkaloids, and Guan-mu-tong (Aristolochia manshuriensis), which contains aristolochic acid (Li et al., 2008; Lai et al., 2009).

Our previous research showed that ZSASW was significantly less toxic than cinnabar (Wang et al., 2013). However, the potential for each herb to alleviate the toxicity of cinnabar still needs to be studied. In the current study, ^1^H NMR spectroscopy was used to analyze the urinary and blood metabolites in rats to investigate the detoxification of each herb in ZSASW. This study demonstrated that NMR-based metabonomics analysis could be a promising approach for investigating the detoxification of Chinese medicines and the rationale behind TCM prescriptions.

## 2 Materials and methods

### 2.1 Reagents and samples

2,2,3,3-Deuterotrimethylsilylpropionic acid (TSP) and D_2_O were purchased from Norell, Inc. (United States). Phosphate buffer was prepared by mixing 0.2 M Na_2_HPO_4_ and 0.2 M NaH_2_PO_4_ (pH 7.4; Sigma, St. Louis, MO, United States). Cinnabars, *Coptidis Rhizoma*, *Angelicae Sinensis Radix, Rehmanniae Radix*, and honey fried *Glycyrrhizae Radix* were purchased from Liaoning Tongren Pharmaceutical Co., Ltd. (Shenyang, China) and authenticated by Professor Jincai Lu (Shenyang Pharmaceutical University, China). All traditional Chinese medicine uses raw materials.

### 2.2 Animals and drug administration

All animal experiments were approved by the Experimental Animal Research Committee of Shenyang Pharmaceutical University (Shenyang, China, SYPU-IACUC-GZR2020-0416-203) and experiments were conducted in accordance with the relevant institutional and national guidelines and regulations. A total of 36 male Wistar rats (SPF, weighing 220 ± 20 g; animal license no. SCXK- (military) 2007-004) were obtained from the Experimental Animal Center of Shenyang Pharmaceutical University. After a seven-day acclimatization period, the rats were kept under standard laboratory conditions, including a temperature of 25°C ± 1°C, relative humidity of 45% ± 15%, and a 12-h light/dark cycle. They were housed in individual metabolic cages with *ad libitum* access to food and water. All rats were randomly divided into 6 groups (*n* = 6): control group (injected with water), cinnabar group (treated with cinnabar at dosage of 1.8 g/kg), CAS group (treated with cinnabar and *Angelica sinensis* at a dosage of 1.8 g/kg), CRG group (treated with cinnabar and *Rehmannia glutinosa* Libosch at a dosage of 1.8 g/kg), CCC group (treated with cinnabar and *Coptis chinensis* French at a dosage of 2.7 g/kg), and CGU group (treated with cinnabar and *Glycyrrhiza uralensis* Fisch at a dosage of 0.9 g/kg) (Wei et al., 2008).

### 2.3 Sample collection and pretreatment

Urine samples were collected overnight on dry ice in tubes containing 1 mL of 1% sodium azide (from PM 8:00 to AM 8:00). These samples were immediately frozen and stored at −20°C until they were prepared for NMR spectroscopic analysis. At the end of the experimental period (day nine), all animals were sacrificed, and blood was drawn from the inferior vena cava. Serum was obtained by centrifugation at 14,000 rpm for 10 min at 4°C. All the serum samples were stored at −80°C until further analysis. Livers and kidneys were removed from the rats and immersed in 10% neutral buffered formaldehyde solution (Sigma, St. Louis, MO, United States) for histopathology experiments.

### 2.4 Serum biochemical analysis and histopathology examination

The liver, kidney and intestinal tract tissues were dehydrated, embedded in paraffin, and cut at 5 μm thickness. The sliced sections were stained with hematoxylin and eosin (H&E) and examined via light microscopy (×200). The serum biochemical test included the following parameters: aspartate aminotransferase (AST), alanine aminotransferase (ALT), alkaline phosphatase (ALP), glucose (GLU), creatinine (CREA), triglyceride (TG), total protein (TP), cholesterol (CHO) and carbamide (UREA).

### 2.5 ^1^H NMR spectroscopic measurements of urine and serum samples

Five hundred microliters of a thawed urine sample was mixed with 200 μL of phosphate buffer (0.2 M, pH 7.4) and centrifuged at 8000 rpm for 10 min to remove insoluble material. Five hundred microliters of supernatant was placed into a 5 mm NMR tube containing 50 μL (TSP, 0.1%, w/v) and 60 μL D_2_O. NMR spectral measurements were acquired on a Bruker-Av600 spectrometer at 298 K. Typically, 32 free induction decays (FIDs) were collected into 64 k data points over a spectral width of 12019.23 Hz with a relaxation delay of 3 s and an acquisition time of 2.73 s. The number of scans was 64.

Frozen serum samples were thawed, and 400 μL of serum was mixed with 60 μL of D_2_O and 40 μL of TSP and transferred to 5 mm NMR tubes. TSP acted as an internal standard reference (*δ* 0.00 ppm), and D_2_O was used for the locking signal. ^1^H NMR spectra of these samples were also recorded on a Bruker-Av600 spectrometer at 298 K. The pulse program was a 1D experiment with a T_2_ filter using the Carr‒Purcell‒Meiboom‒Gill sequence. Thirty-two FIDs were collected into 64 k data points over a spectral width of 12019.23 Hz with a relaxation delay of 3 s and an acquisition time of 2.73 s. The power level for presaturation was 50 dB, and the number of scans was also 64.

### 2.6 Data reduction analysis of ^1^H NMR spectra

Baseline-corrected and manual phase adjustments of the spectra were performed. The ^1^H NMR spectra from urine or serum were segmented into 192 integrated regions of equal width (0.04 ppm), corresponding to the region *δ* 0.2–10.0, using MestReNova 9.0.1 (Mestrelab Research SL.). The area for each segmented region was calculated, and the integral values contributed to an intensity distribution of the whole spectrum. The region (*δ* 4.2–5.0) was deleted to remove any spurious effects of variability in the suppression of water resonance. For the urine spectra, the region containing urea (*δ* 5.2–6.0) was also excluded to eliminate any consequent chemical exchange effects on the urea signal. All remaining regions of the spectra were then scaled to the total integrated area of the spectra to reduce any significant concentration differences.

Finally, all the spectral data were normalized to a constant integrated intensity. The data set was mean centered, meaning that the average peak intensity across all samples was set to zero. PLS-DA-based multivariate data analysis was performed using the SIMCA-P 13.0 software package (Umetrics AB, Umea, Sweden).

The goodness of fit of each model was evaluated using three quantitative parameters: R^2^X was the explained variation in X, R^2^Y was the explained variation in Y, and Q^2^Y was the predicted variation in Y. The range of these parameters was between 0 and 1, with values approaching 1 indicating a perfect fit of the model. (Liu et al., 2010).

## 3 Results

### 3.1 Clinical biochemistry and histopathology

To verify the presence of liver damage in each group of rats, the serum levels of AST, ALT, ALP, TP, UREA, CREA, TG, CHO, and GLU were measured. The biochemical changes in the serum are presented in Table 1. Significantly increased levels of serum AST and ALP were found in the cinnabar and CRG groups compared with those in the control group. However, there were no significant changes in the levels of urea, glucose or creatinine among the CCC and CGU groups.

**TABLE 1:** Selected clinical biochemical parameters of serum. The data are presented as the means ± SDs of six animals per group.

Biochemical parameters	Control	Cinnabar	CAS	CRG	CGU	CCC
AST (U/L)	94.50 ± 19.07	115.17 ± 16.24^a^	80.60 ± 13.97	106.50 ± 16.22	96.83 ± 26.24	94.67 ± 16.12
ALT (U/L)	34.00 ± 9.01	39.00 ± 8.72	32.40 ± 5.64	41.67 ± 2.80^a^	35.50 ± 5.50	39.33 ± 8.09
ALP (U/L)	262.83 ± 105.27	293.17 ± 47.82^a^	253.80 ± 74.34	314.17 ± 56.39^a^	283.17 ± 80.02	267.17 ± 54.55
TP (g/L)	61.58 ± 11.94	71.62 ± 6.74	63.56 ± 11.43	77.35 ± 7.78^a^	65.68 ± 15.41	67.68 ± 15.76
UREA (lmol/L)	12.88 ± 3.70	10.87 ± 0.86	10.74 ± 1.67	12.05 ± 2.05	10.47 ± 2.51^b^	10.58 ± 1.61
CREA (lmol/L)	20.00 ± 4.86	18.17 ± 3.43	17.00 ± 2.83^a^	20.83 ± 5.04	18.17 ± 6.24^b^	17.83 ± 3.06^b^
TG (mmol/L)	0.64 ± 0.21	0.72 ± 0.22	0.62 ± 0.30	0.62 ± 0.20	0.52 ± 0.11	0.45 ± 0.13
CHO (mmol/L)	1.63 ± 0.46	1.55 ± 0.34	1.50 ± 0.34^a^	1.62 ± 0.15	1.30 ± 0.59^b^	1.23 ± 0.33
GLU (mmol/L)	12.79 ± 2.44	14.17 ± 1.47	13.97 ± 2.22^a^	15.79 ± 1.57^a^	13.05 ± 3.44	13.64 ± 2.06

^a^ *p* < 0.05 compared to the control.

^b^ *p* < 0.05 compared to cinnabar.

**FIGURE 1:**
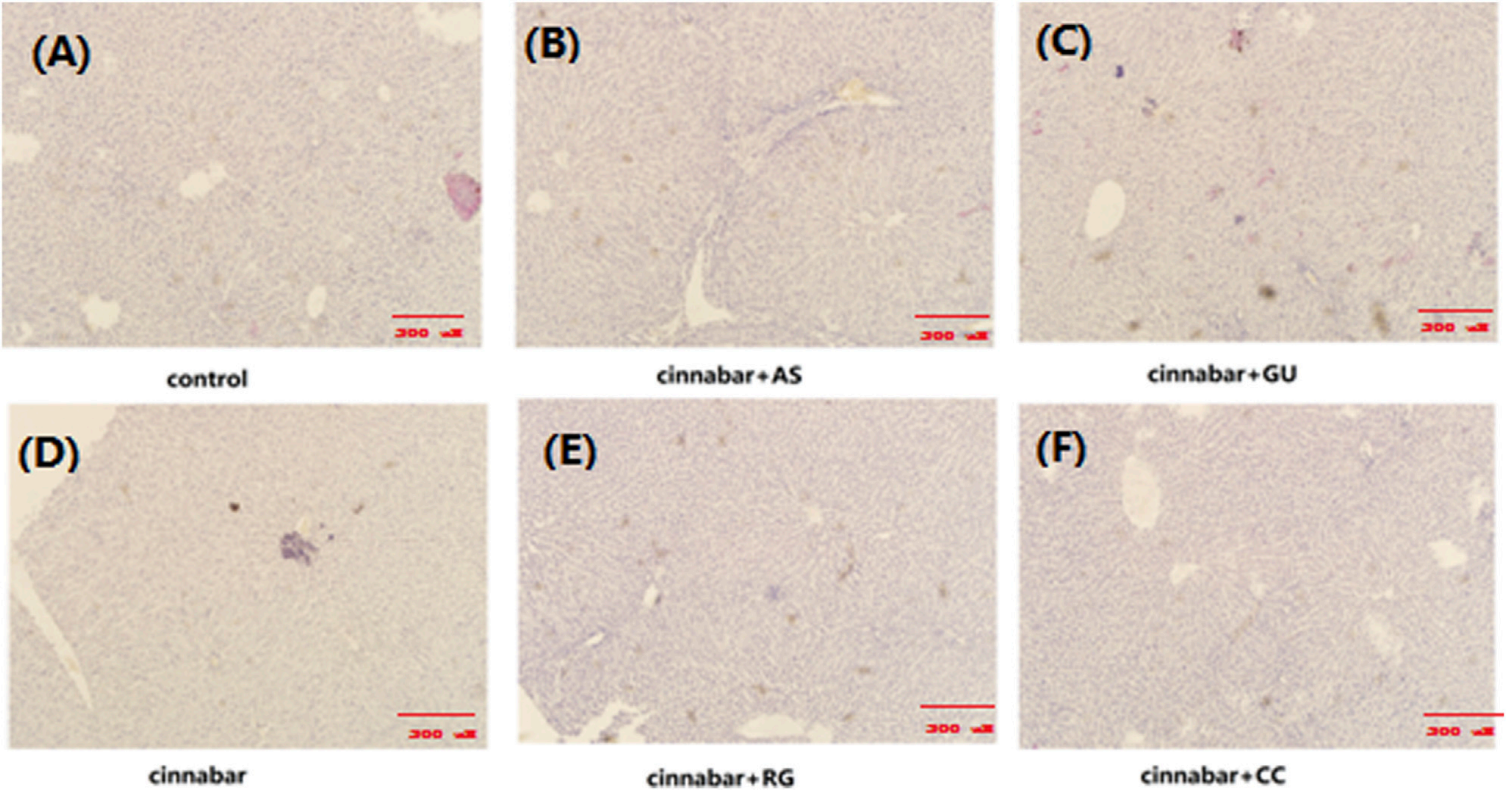
Liver histopathology of rats. Livers from the control **(A)**, CAS **(B)**, CGU **(C)**, cinnabar **(D)**, CRG **(E)**, and CCC **(F)** groups.

**FIGURE 2:**
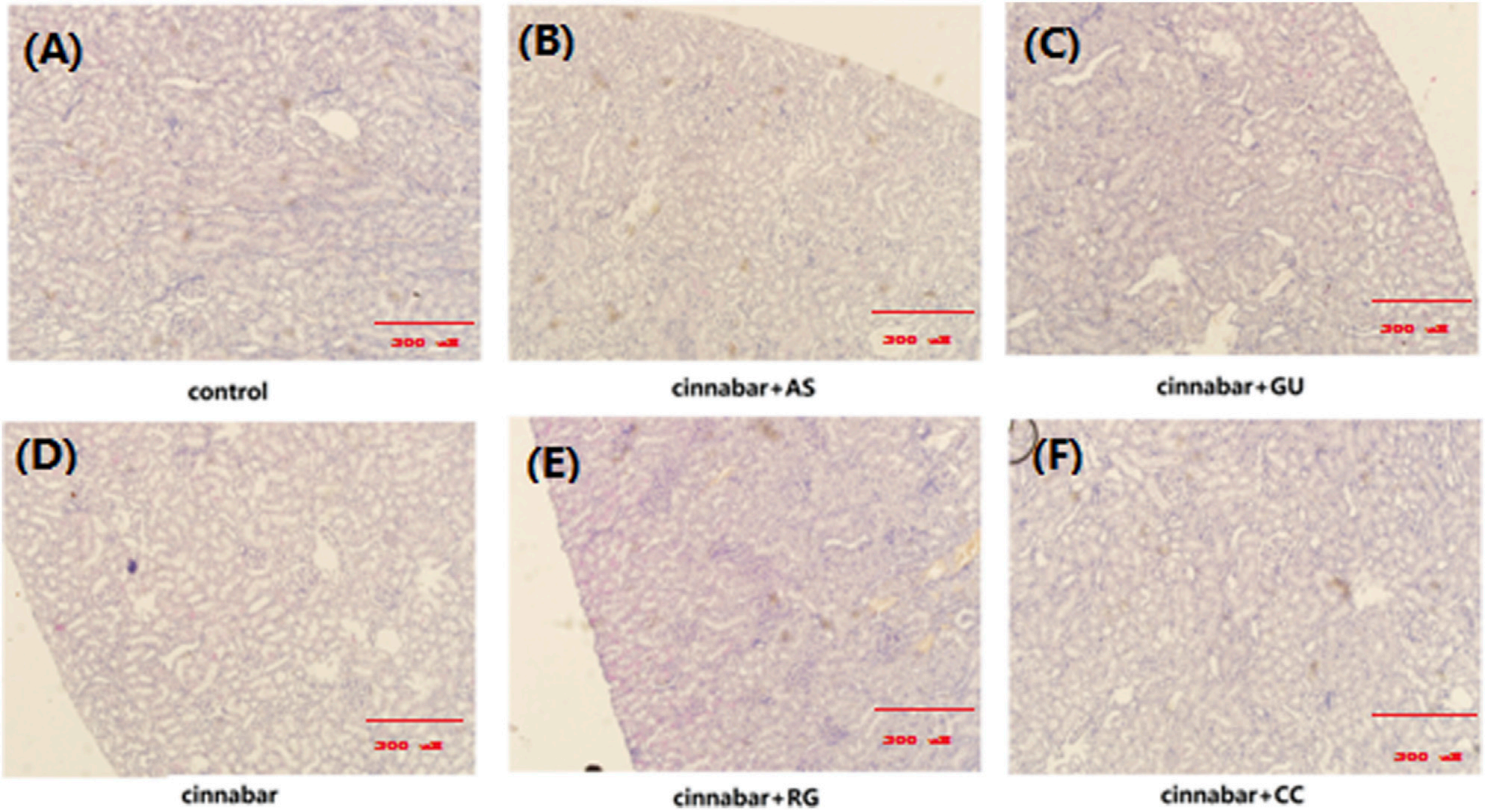
Kidney histopathology of rats. Kidneys from the control **(A)**, CAS **(B)**, CGU **(C)**, cinnabar **(D)**, CRG **(E)**, and CCC **(F)** groups.

As demonstrated in Figure 1, the main lesions in the cinnabar and CAS groups exhibited diffuse hepatocyte degeneration, necrosis, and apoptosis. Significant swelling (ballooning degeneration) (Figure 2) of hepatocytes was also observed in the cinnabar, CAS and CRG groups. On the other hand, no liver or renal damage was observed in the control group, CCC group or CGU group.

**FIGURE 3:**
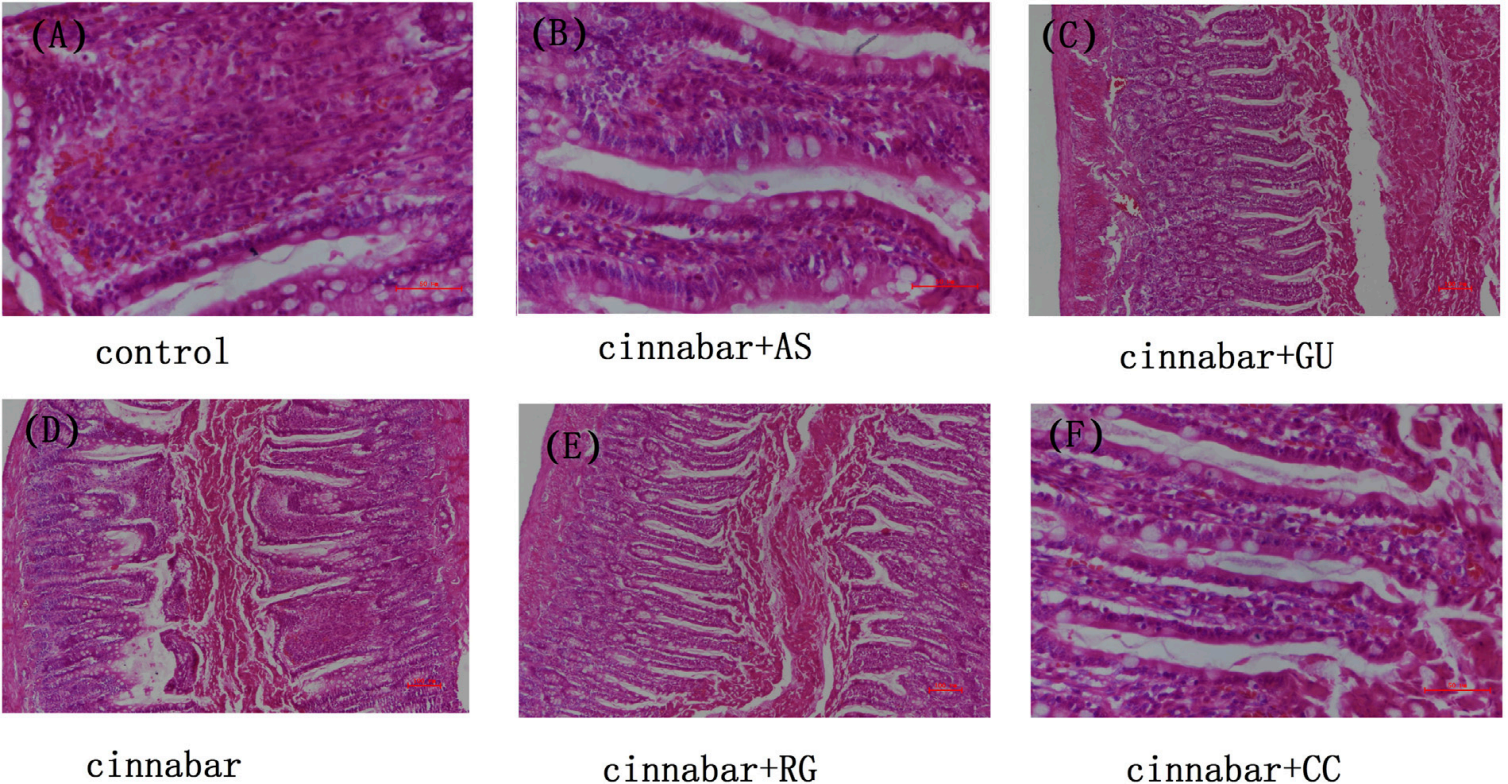
Intestinal histopathology of rats. Kidneys from the control **(A)**, CAS **(B)**, CGU **(C)**, cinnabar **(D)**, CRG **(E)**, and CCC **(F)** groups.

After the administration of cinnabar, most red blood cells in the intestinal cavity of the rats appeared as blood. Most inflammatory cells can be observed in the intestinal chorion. No abnormalities were found in the intestinal cavity or chorionic membrane of the other administration groups, including the control group (Figure 3). The above results indicate that other Chinese herbs in Zhusha Anshenwan may have potential protective effects on intestinal injury caused by cinnabar.

### 3.2 Analysis of ^1^H NMR spectral data of urine

**FIGURE 4:**
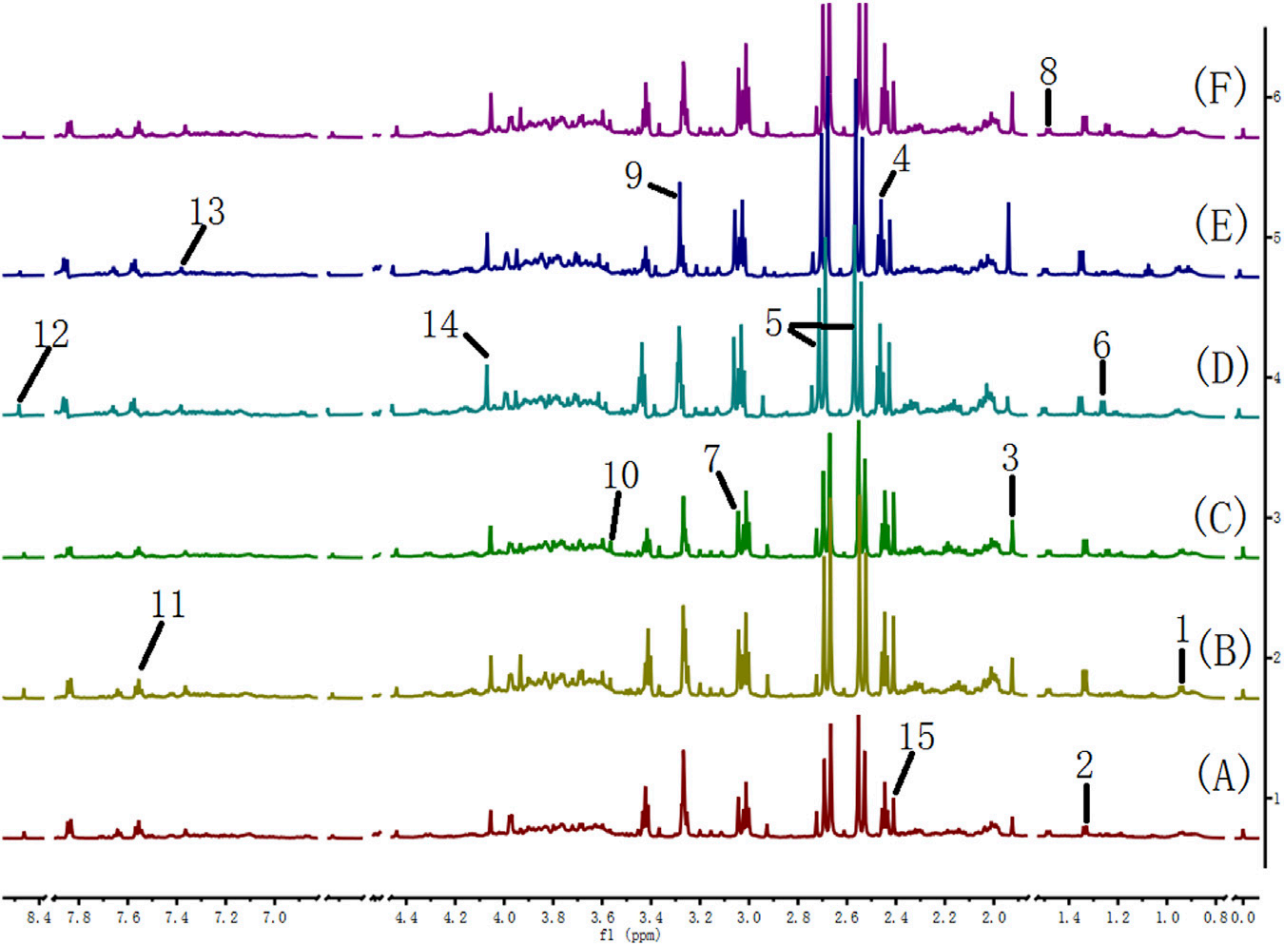
600 MHz ^1^H NMR spectra of urine from the cinnabar group **(A)**, control group **(B)**, CCC group **(C)**, CAS group **(D)**, CRG group **(E)**, and CGU group **(F)**. Keywords: 1, leucine + isoleucine; 2,3-hydroxybutyrate (3-HB); 3, acetate; 4,2-ketoglutarate (2-OG); 5, citrate; 6, lactate; 7, creatine; 8, alanine; 9, trimethylamine-*N*-oxide (TMAO); 10, glycine; 11, hippurate; 12, formate; 13, phenylalanine; 14, creatinine; 15, succinate; 16, taurine.

**FIGURE 5:**
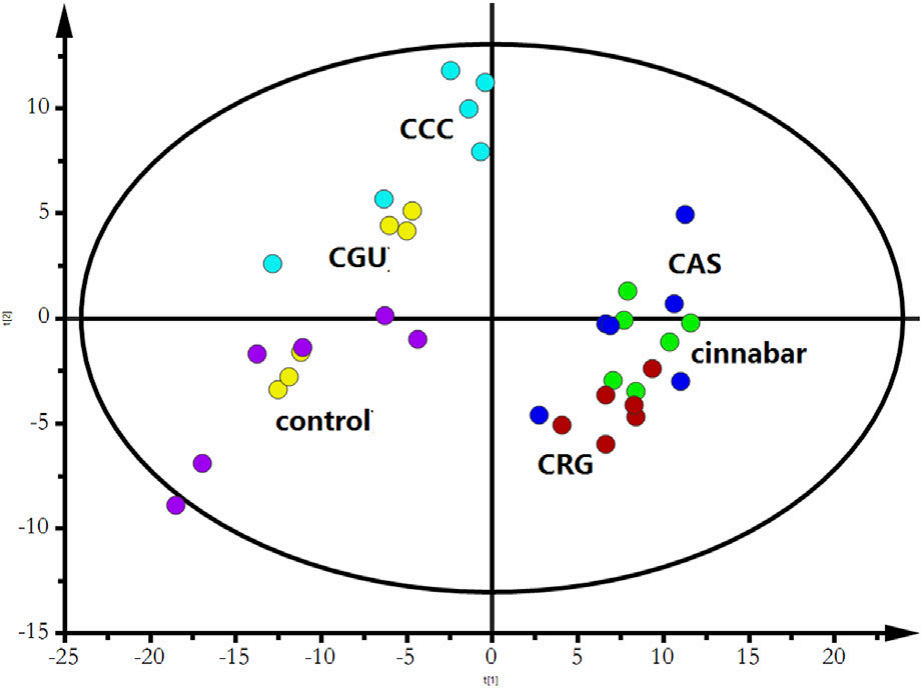
PLS-DA score plot derived from ^1^H NMR spectra of urine from the 6 groups (R^2^X = 0.453, Q^2^ = 0.318). Keywords: control group (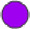); CCC group (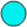); CGU group (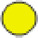); cinnabar group (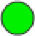); CAS group (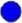); CRG group (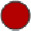).

Many endogenous metabolites were observed in the ^1^H NMR spectra of urine from the control, cinnabar, CAS, CGU, CRG, and CCC groups on day 9 (Figure 4). Assignments of endogenous metabolites involved in ^1^H NMR spectra were based on the literature (Jung et al., 2011; Tang et al., 2012). The main endogenous metabolites, such as leucine, isoleucine, 3-hydroxybutyrate (3-HB), acetate, 2-ketoglutarate (2-OG), citrate, lactate, creatine, alanine, trimethylamine-*N*-oxide (TMAO), glycine, hippurate, formate, phenylalanine, creatinine and succinate, were identified (Figure 4). To illustrate the differences in the metabolic profiles, a PLS-DA score plot based on ^1^H NMR spectra of urine from the 6 groups is shown in Figure 5.

**FIGURE 6:**
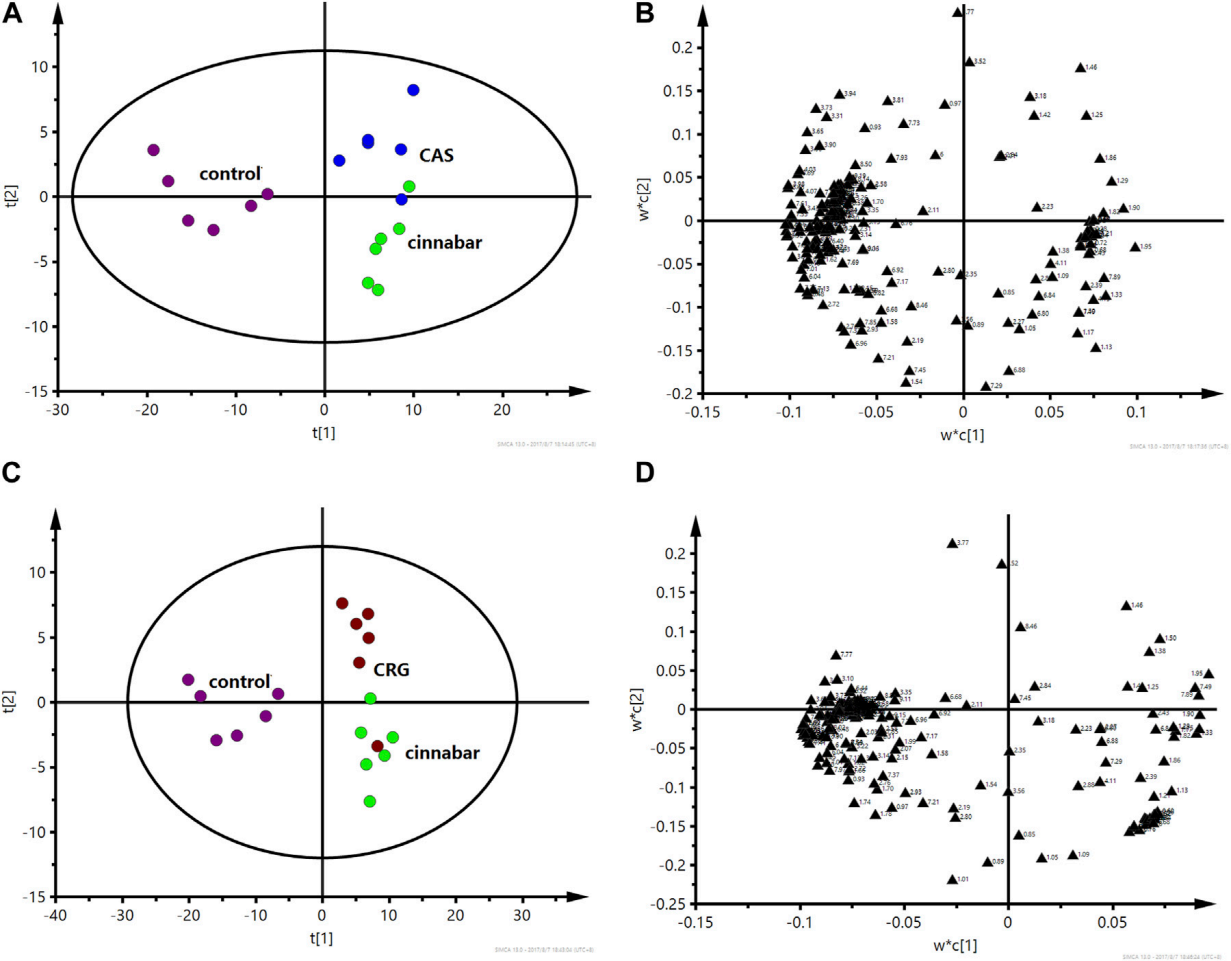
PLS-DA score plot **(A)** and corresponding loading plot **(B)** based on ^1^H NMR spectra of urine from the control group, cinnabar group and CAS group (R^2^X = 0.545, Q^2^ = 0.352). PLS-DA score plot **(C)** and corresponding loading plot **(D)** based on ^1^H NMR spectra of urine from the control group, cinnabar group and CRG group (R^2^X = 0.577, Q^2^ = 0.353). Key: control group (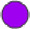); group CAS (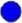); group CRG (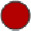); group cinnabar (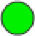).

**FIGURE 7:**
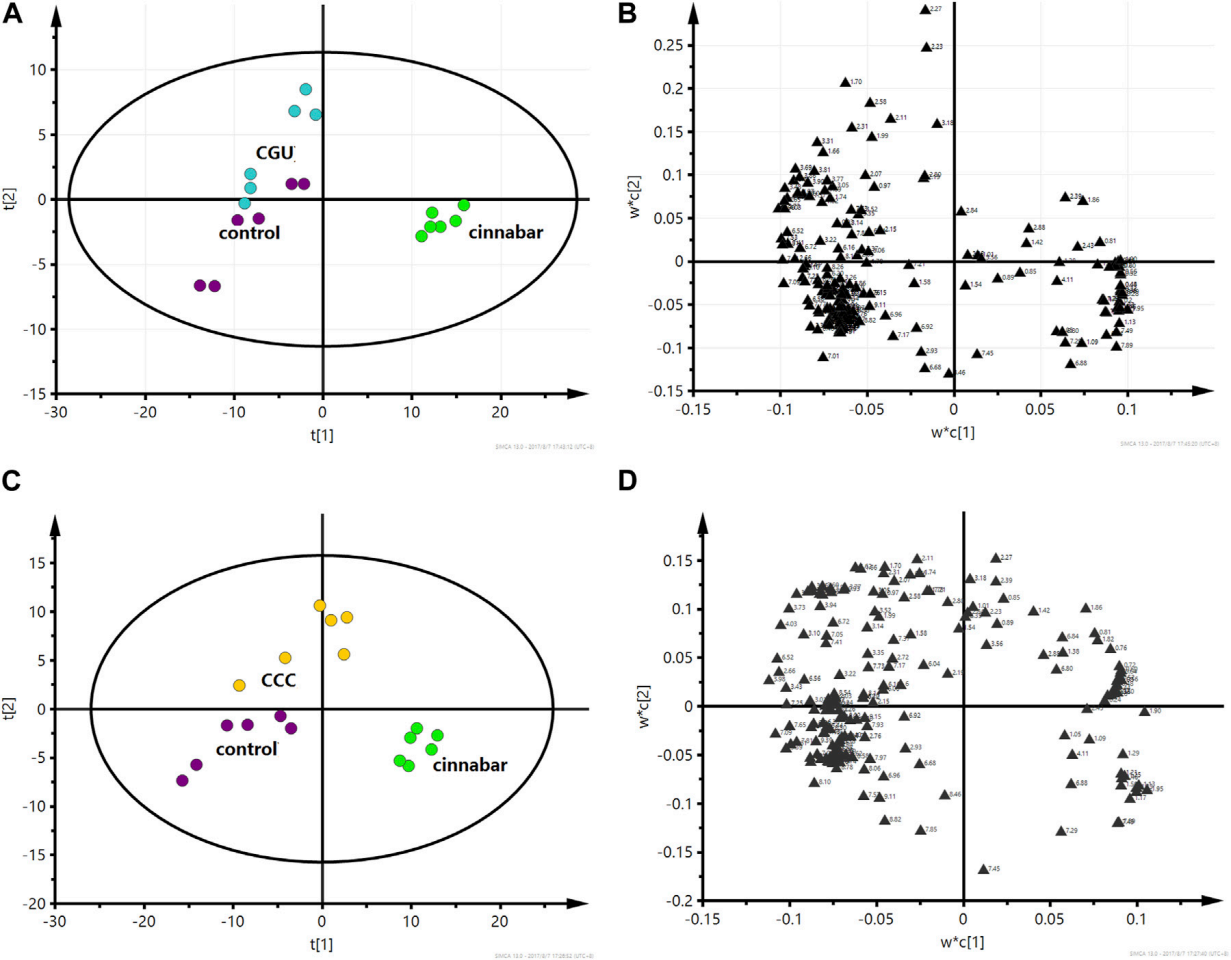
PLS-DA score plot **(A)** and corresponding loading plot **(B)** based on ^1^H NMR spectra of urine from the control group, cinnabar group and CGU group (R^2^X = 0.557, Q^2^ = 0.428). PLS-DA score plot **(C)** and corresponding loading plot **(D)** based on ^1^H NMR spectra of urine from the control group, cinnabar group and CCC group (R^2^X = 0.466, Q^2^ = 0.324). Key: control group (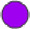); group CGU (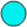); group CCC (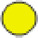); group cinnabar (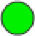).

As shown in Figure 5, the PLS-DA results, as scores for the first two principal components from the spectra of the six groups, revealed that the CGU and CCC groups were distributed in the area of the control group, while the CAS and CRG groups were close to the cinnabar group. To investigate the impact of each herb in ZSASW on metabolic changes in detail, comparisons among the control and compatibility groups of cinnabars (CAS, CRG, CCC, CGU) were carried out using the PLS-DA model (Figures 6, 7).

In the PLS-DA-derived score plots (Figures 6A, C), the clusters of the cinnabar and CAS groups as well as the CRG were easily distinguished from those of the control group. The loading plots (Figures 6B, D) showed the relevant changes in endogenous metabolites responsible for the separation, which included increased alanine, lactate, TMAO, taurine, hippurate, phenylalanine, 3-HB, creatine and creatinine and decreased 2-OG, succinate, and citrate in the cinnabar, CAS and CRG groups compared with those in the control group.

However, from the PLS-DA score plots (Figures 7A, C), separation of the cinnabar group from the control, CGU and CCC groups was observed. By examining the corresponding loading plots (Figures 7B, D) and NMR spectra, the separation was attributed to depleted alanine, lactate, TMAO, phenylalanine, 3-HB, creatine and creatinine with elevated citrate, succinate, 2-OG, and hippurate in the CGU and CCC groups compared to the cinnabar group.

The endogenous metabolites in urine samples and their changes in different groups based on the analyses of the loading plots and the ^1^H NMR-detected relative integral levels are listed in Table 2.

**TABLE 2:** Endogenous metabolites selected as biomarkers in urine profiles and their change trends.

Endogenous metabolites	Chemical shifts	Cinnabar VS Control	CAS VS Cinnabar	CRG VS Cinnabar	CGU VS Cinnabar	CCC VS Cinnabar
citrate	2.72 (d), 2.56 (d)	↓	↑	↓	↑	↑
TMAO	3.26 (s)	↑	↑	↓	↓	↓
succinate	2.42 (s)	↓	↓	↑	↑	↑
2-OG	3.01 (t), 2.45 (t)	↓	↓	↑	↑	↑
hippurate	7.73 (d), 7.64 (t), 7.55 (t)	↓	↑	↑	↑	↑
	3.97 (d)					
formate	8.47 (s)	↓	↑	↑	↑	↑
taurine	3.43 (t), 3.26 (t)	↑	↑	↓	↑	↑
creatine	3.94 (s), 3.04 (s)	↑	↑	↑	↓	↓
creatinine	4.05 (s), 3.05 (s)	↑	↑	↑	↓	↓
phenylalanine	7.37 (m), 7.42 (m)	↑	↑	↑	↓	↓
lactate	4.11 (q), 1.33 (d)	↑	↓	↓	↓	↓
glycine	3.55 (s)	↑	↓	↓	↓	↓
3-HB	1.23 (d)	↑	↑	↓	↓	↓
alanine	1.48 (d)	↑	↑	↓	↓	↓
acetate	1.92 (s)	↑	↓	↑	↓	↓

The levels of potential biomarkers are labeled with (↓) indicating a decrease and (↑) indicating an increase.

### 3.3. Analysis of ^1^H NMR spectral data of serum

**FIGURE 8:**
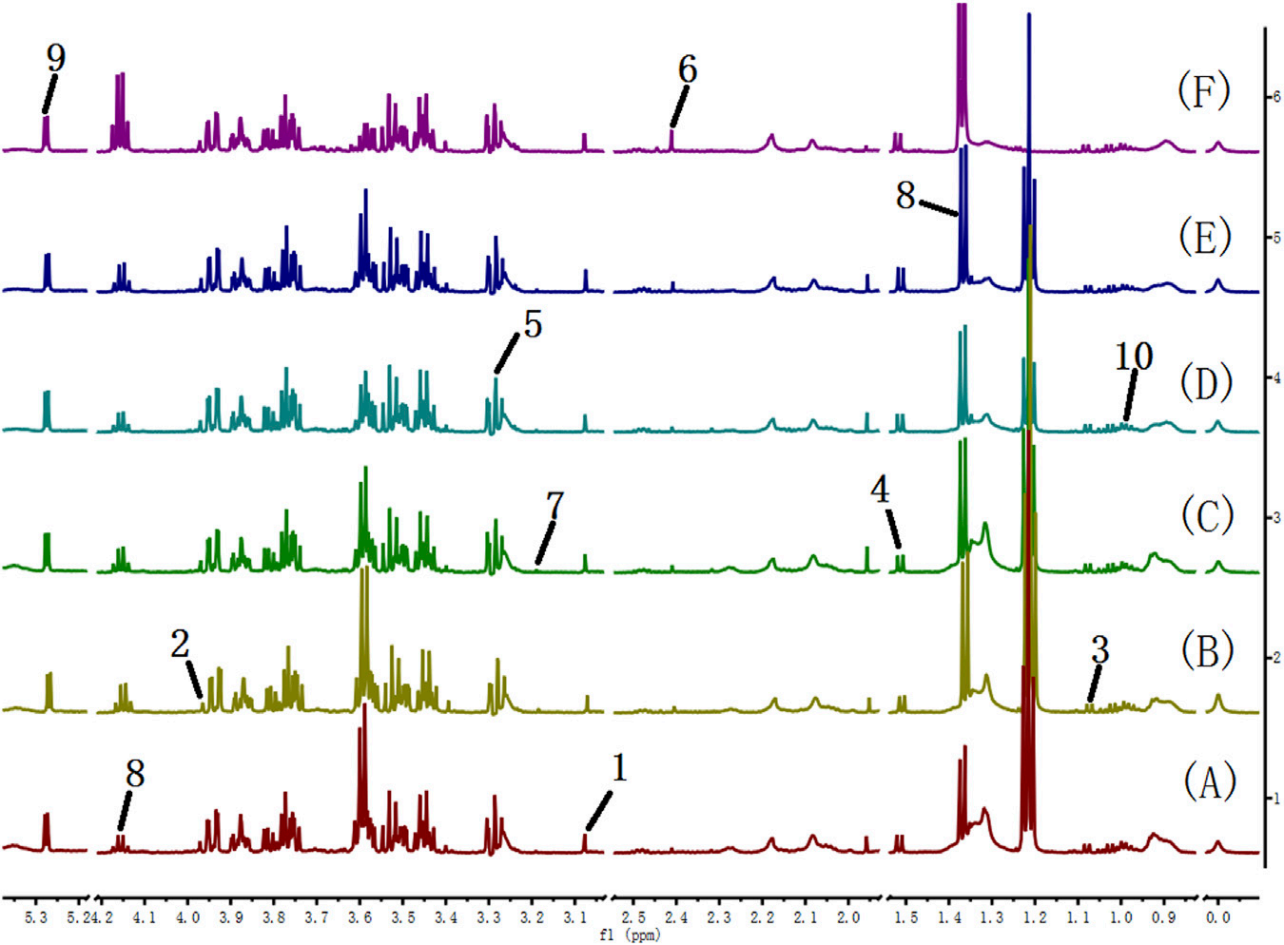
600 MHz ^1^H NMR spectra of serum from the cinnabar group **(A)**, control group **(B)**, CCC group **(C)**, CAS group **(D)**, CRG group **(E)**, and CGU group **(F)**. Key: 1, creatinine; 2, creatine; 3, valine; 4, alanine; 5, TMAO; 6, pyruvate; 7, choline; 8, lactate; 9, *α*-glucose; 10, leucine + isoleucine; 11, acetate.

The ^1^H NMR spectra of the serum of rats in different groups are shown in Figure 8. The metabolite signals that were assigned to the proton region of the ^1^H NMR spectra included those corresponding to creatinine, creatine, valine, alanine, TMAO, pyruvate, choline, lactate, *α*-glucose, leucine and isoleucine (Nicholls et al., 2001).

**FIGURE 9:**
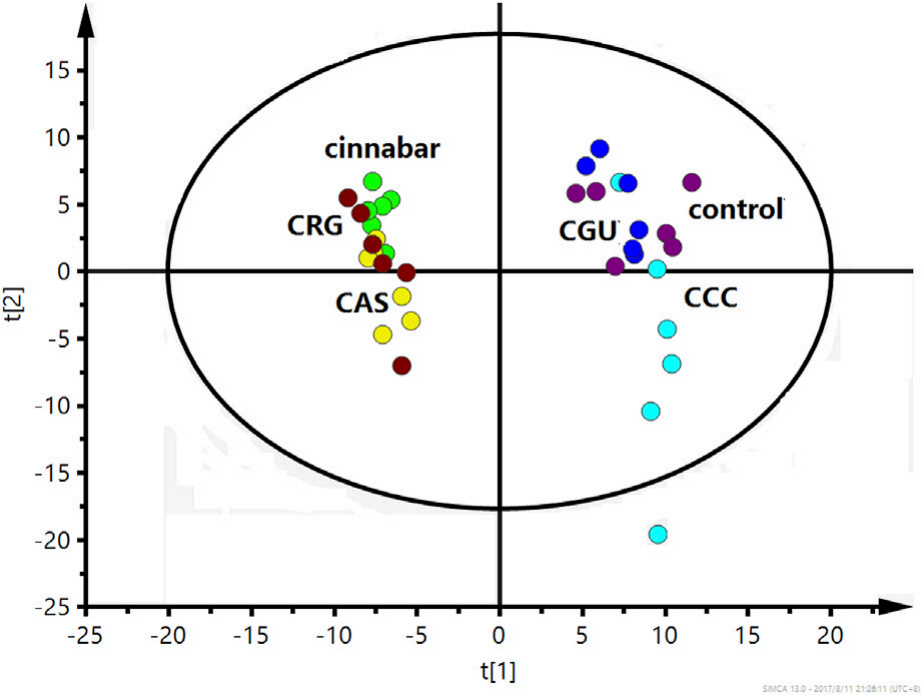
PLS-DA score plot derived from ^1^H NMR spectra of serum from the 6 groups (R^2^X = 0.682, Q^2^ = 0.598). Key: control group (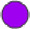); group CCC (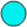); group CAS (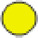); group cinnabar (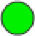); group CGU (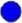); group CRG (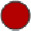).

The PLS-DA score plot based on ^1^H NMR spectra of the serum from the six groups is shown in Figure 9. The points of the CCC and CGU groups were classified to the region of the control group, while the cinnabar, CAS and CRG groups were mapped together.

**FIGURE 10:**
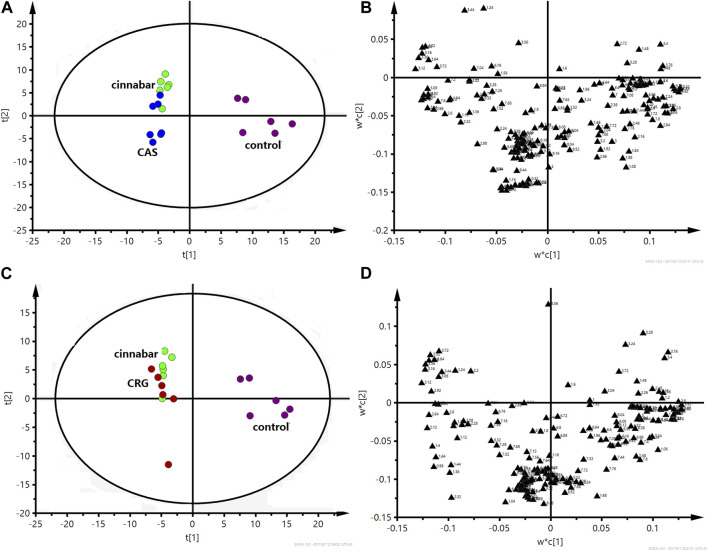
PLS-DA score plot **(A)** and corresponding loading plot **(B)** based on ^1^H NMR spectra of serum from the control group, cinnabar group and CAS group (R^2^X = 0.545, Q^2^ = 0.352). PLS-DA score plot **(C)** and corresponding loading plot **(D)** based on ^1^H NMR spectra of serum from the control group, cinnabar group and CRG group (R^2^X = 0.577, Q^2^ = 0.353). Key: control group (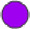); group CAS (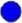); group CRG (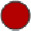); group cinnabar (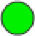).

The PLS-DA score plots (Figures 10A, C) showed that the cinnabar, CAS and CRG groups were well discriminated from the control group. The loading plot (Figures 10B, D) revealed that the difference was attributed to an increase in the levels of creatinine, creatine, valine, alanine, TMAO, leucine and isoleucine as well as a reduction in the level of lactate in the cinnabar, CAS and CRG groups compared with the control group.

**FIGURE 11:**
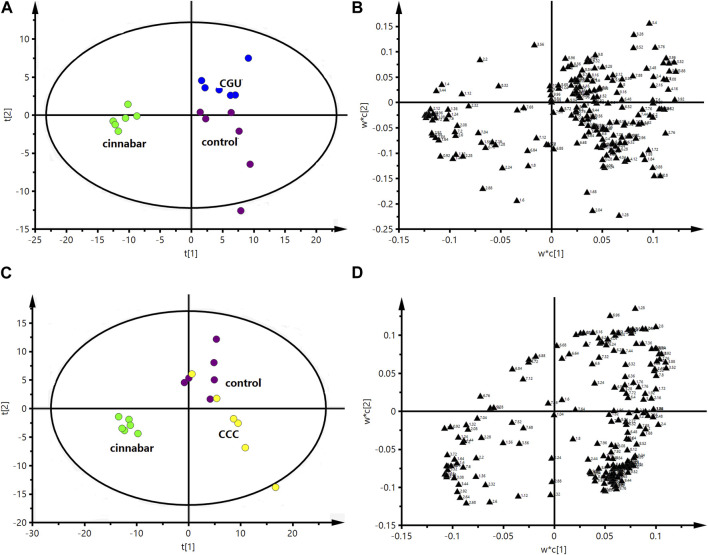
PLS-DA score plot **(A)** and corresponding loading plot **(B)** based on ^1^H NMR spectra of serum from the control group, cinnabar group and CGU group (R^2^X = 0.508, Q^2^ = 0.412). PLS-DA score plot **(C)** and corresponding loading plot **(D)** based on ^1^H NMR spectra of serum from the control group, cinnabar group and CCC group (R^2^X = 0.466, Q^2^ = 0.324). Key: control group (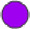); group CCC (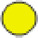); group CGU (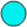); group cinnabar (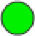).

The performance of the score plots (Figures 11A, C) reflected discrimination where the control, CCC and CGU groups were separate from the cinnabar group. The corresponding loading plots (Figures 11B, D) and ^1^H NMR spectra showed that the main biochemical changes in the serum were decreased creatinine, creatine, valine, alanine, TMAO, leucine and isoleucine with increased choline, pyruvate and lactate in the control, CCC and CGU groups compared with the cinnabar group.

The assigned metabolites and their relative variations in serum samples from the different groups according to the PLS-DA loading plots and the ^1^H NMR-detected relative integral levels of metabolites are listed in Table 3.

**TABLE 3:** Endogenous metabolites selected as biomarkers in the serum profile and their change trends.

Endogenous metabolites	Chemical shifts	Cinnabar VS Control	CAS VS Cinnabar	CRG VS Cinnabar	CGU VS Cinnabar	CCC VS Cinnabar
creatinine	4.05 (s), 3.05 (s)	↑	↑	↑	↓	↓
creatine	3.94 (s), 3.04 (s)	↑	↑	↑	↓	↓
valine	0.98 (d), 1.02 (d)	↑	↑	↓	↓	↓
alanine	1.48 (d)	↑	↓	↑	↓	↑
TMAO	3.26 (s)	↑	↑	↓	↓	↓
pyruvate	2.38 (s)	↓	↑	↑	↑	↑
choline	3.18 (s)	↓	↑	↑	↑	↑
lactate	4.11 (q),1.33 (d)	↓	↓	↓	↑	↑
leucine + isoleucine	0.96–1.0 (m)	↑	↑	↓	↓	↓

The levels of potential biomarkers are labeled with (↓) indicating a decrease and (↑) an increase.

### 3.4 Pathway analysis

**FIGURE 12:**
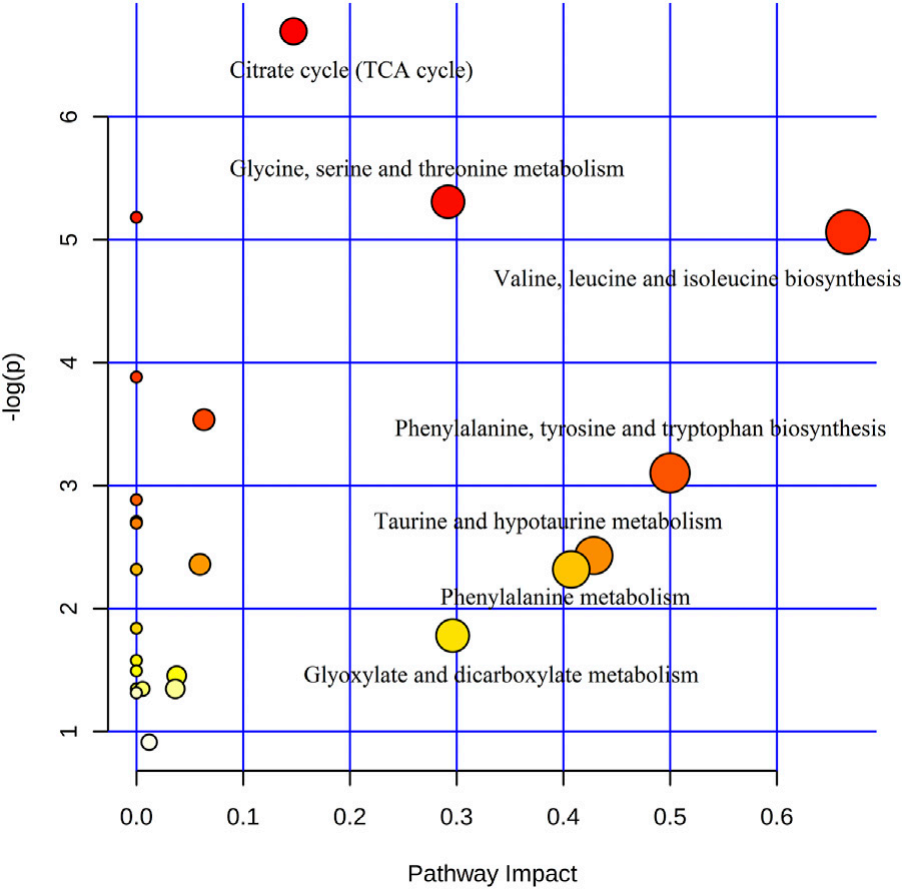
Summary of pathway analysis.

In addition, to identify the most relevant pathways associated with cinnabar-induced acute hepatic injury, a comprehensive metabolic network was constructed using MetaboAnalyst 3.0. A total of sixteen biomarkers underwent pathway analysis via metaboanalyst 3.0 (http://www.metaboanalyst.ca), and the metabolic pathways associated with each substance and their FDR values are summarized in Figure 12. Pathways with an impact value >0.1 were considered the most relevant pathways to liver injury. In this study, the biosynthesis of valine, leucine, and isoleucine; as well as the metabolism of taurine and hypotaurine, phenylalanine, glycine, serine, and threonine, glyoxylate and dicarboxylate, and the TCA cycle pathways were identified as important metabolic pathways, with impact factors of 0.67, 0.42 0.41, 0.29, 0.29, and 0.15, respectively. Pathway analysis indicated that the alleviation effect of each herb in ZSASW was connected to alterations in energy metabolism and amino acid metabolism.

## 4 Discussion

Our previous research investigated the liver and kidney toxicity of cinnabar. However, at the toxic dose, no corresponding damage was detected in the zhusha Anshen wan. We speculate that each single traditional Chinese medicine in the formula has a certain protective effect on the damage caused by cinnabar. Based on the relationship of the various traditional Chinese medicine combinations in the formula, in order to provide a better basis for the rational clinical use of the formula, we conducted a preliminary exploration of the detoxification effect of each single drug on cinnabar in the Zhusha Anshen wan.

Succinate, citrate, and 2-OG are the three key intermediates in the TCA cycle. The TCA cycle is a series of chemical reactions that breakdown glucose into energy molecules such as ATP. Downregulation of the TCA cycle may lead to a reduction in the levels of cinnabar and CAS groups. The tricarboxylic acid (TCA) cycle is a metabolic pathway that converts glucose into energy molecules, including ATP (Martínez-Reyes et al., 2016). Succinate is converted back to citric acid, which is then further converted to CO_2_ and used for respiration. However, succinate, citrate, and 2-OG tended to reverse the changes observed in the CCC and CGU groups, indicating that the herbs *Coptis chinensis* and *Glycyrrhiza uralensis* Fisch could reverse energy metabolism.

On the other hand, lower ATP disrupts mitochondrial function, leading to higher AMP concentrations. We observed high levels of alanine, an intermediate of anaerobic metabolism, in the serum of the cinnabar and CRG groups. An increase in amino acids, such as alanine and phenylalanine, was also observed in the rats treated with cinnabar, CAS, or CRG, which indicated disruption of hepatic amino acid metabolism. This could have resulted from hepatic damage or depletion of hepatic ATP induced by cinnabar treatment. Elevated levels of branched-chain amino acids (leucine and valine) were observed in the serum of cinnabar and CAS groups. A notable rise in alanine in the serum was also observed after cinnabar or CRG treatment. This finding implies that cinnabar disrupts hepatic amino acid metabolism. It is also thought that the loss of absorption capacity of the proximal tubules by cinnabar increases the excretion of amino acids such as alanine, isoleucine, leucine and valine. Moreover, the herbs *Coptis chinensis* and *Glycyrrhiza uralensis* Fisch reversed the increase in alanine, phenylalanine and lactate in the urine, which suggested that these two herbs could prevent liver and kidney damage induced by cinnabar.

It appears that betaine primarily affects peripheral tissues. Additionally, it has been reported that TMAO in plasma is produced through the following pathway: dietary phosphatidylcholine/choline → gut flora-formed TMA → hepatic FMO-formed TMAO (Feng et al., 2014), which was elevated in the rats in the cinnabar and CAS groups. The development of hydropic degeneration due to the hypotonic conditions of the extracellular fluid could associate with elevated levels of TMAO and betaine. A high level of urinary TMAO (an osmolyte mentioned above), commonly associated with osmotic stress in the renal medulla and a signal of drug-induced nephrotoxicity (Feng et al., 2002), supporting evidence for renal medullary damage, could also be found from the exfoliated epithelial cells and proteins in the lumen of the renal medulla as noted in the histopathological analysis. *Rehmannia glutinosa*, *Rhizoma Coptidis* and *Glycyrrhiza uralensis* Fisch reversed the increase in urine TMAO concentration induced by cinnabar, which indicated that these three herbal medicines might have protective effects on cinnabar-induced renal injury.

Taurine possesses numerous essential properties, including antioxidant effects, regulation of Ca^2+^ flux, membrane stabilization, osmoregulation, and attenuation of apoptosis (Rashid et al., 2013). Increased taurine levels have long been recognized as a specific indicator of liver toxicity in the urine (Waterfield et al., 1991). In the current study, the urinary taurine concentration was elevated in the cinnabar and CAS groups. These changes were accompanied by necrosis and steatosis, which correlated with the observed hepatocyte necrosis in histopathology. Furthermore, the significant increase in the serum AST concentration also provided evidence of hepatic injury. However, the taurine content was reduced by *Rehmannia glutinosa* in the CRG group. The level of taurine in the CRG was close to that in the control group. These results suggest that *Rehmannia glutinosa* could regulate the disruption of taurine metabolism caused by cinnabar.

Additionally, the urinary excretion of creatine and creatinine significantly increased, indicating that cinnabar impaired glomerular filtration function. Histopathological examination revealed evident kidney injury and dysfunction, characterized by vascular dilatation and congestion in the nephron. Additionally, elevated plasma creatinine levels were observed in the biochemical analysis. The fluctuations in biomarker concentrations and histopathology results indicated that *Coptis chinensis* and *Glycyrrhiza uralensis* in ZSASW were the main detoxification materials used for the liver and kidney damage induced by cinnabar.

*Coptis chinensis* (Huanglian) is a commonly used traditional Chinese medicine (TCM) herb, and alkaloids are the most important chemical constituents (Sun et al., 2014; Dai et al., 2016). Alkaloids reportedly have protective effects on the liver (Heidari et al., 2017; Wu et al., 2017). Furthermore, berberine, which is the main alkaloid in *Coptis chinensis*, could restore normal hepatic mitochondrial respiration (Teodoro et al., 2013). Analysis of the metabolic pathways associated with endogenous metabolites revealed that energy metabolism was involved in the detoxification mechanism. Therefore, one of the material criteria for the detoxification of cinnabars might be the presence of alkaloids in *Coptis chinensis*. *Glycyrrhiza uralensis* is one of the most popular herbal medicines worldwide and contains a large array of saponins (Tao et al., 2013; Farag et al., 2015). The pharmacological effects of *Glycyrrhiza uralensis* include the inhibition of gastric ulcers; anti-inflammatory, antiviral, and therapeutic effects on liver ailments have been well verified (Farag et al., 2015; Qiao et al., 2015; Yuan et al., 2016). The biological effects are attributed to the major biologically active constituents: terpenoids, alkaloids and saponins (Zhang and Ye, 2009). Glycyrrhizic acid (GA) is a natural constituent isolated from the dried roots of the genus *Glycyrrhiza*. GA has a potent protective effect against hepatotoxicity. Pretreatment with GA significantly decreased ALT, AST and ALP, attenuated the histopathology of hepatic injury, decreased the apoptotic index, ameliorated oxidative stress in hepatic tissue, and increased the activities of SOD and GPx (Orazizadeh et al., 2014). In combination with our experimental results, we concluded that *Glycyrrhiza uralensis* may have a detoxifying effect on cinnabar-induced injury, probably because it contains saponins, especially larger amounts of glycyrrhizic acid.

Choline can be broken down by the intestinal microbiota into methylamine metabolites, including DMA, TMA, and TMAO. The level of TMAO in the urine of cinnabar-treated rats was elevated, indicating that the choline metabolism of the intestinal microbiota accelerated under the pressure of cinnabar. *Rehmannia glutinosa*, *Rhizoma Coptidis* and *Glycyrrhiza uralensis* Fisch could reverse the increase in urine TMAO concentration induced by cinnabar, which indicated that these three kinds of herbal medicine might regulate intestinal disorders caused by cinnabar and maintain the dynamic balance of the intestinal flora. In addition, hipp is an important metabolite of amino acids in the intestinal microbiota. Hipps are formed by the combination of glycine and benzoate in liver tissue.

## 5 Conclusion

The results clearly showed that the metabolic profiles of the CCC and CGU groups were remarkably similar to those of the control group. The variation tendencies of the CAS and CRG groups were similar to that of the cinnabar group. In this study, the effects of the main detoxification herbs in ZSASW on liver and kidney damage induced by cinnabar were investigated using ^1^H NMR-based metabonomics for the first time. ^1^H NMR spectroscopy, along with histopathology and clinical biochemical assays, provided valuable insight into the relationship between metabolite changes and tissue toxicity. The associated biochemical pathways related to energy metabolism, amino acid metabolism, and gut microbiota disorders offer new insights for assessing the detoxification of each herb in ZSASW from a comprehensive and systematic perspective.

## Data Availability

The data presented in the study are available within the article or supplementary material.
